# Wave Measurements Using Multi-Frame Processing of Marine Radar Data

**DOI:** 10.3390/s21165639

**Published:** 2021-08-21

**Authors:** David Lyzenga, Mirko Previsic

**Affiliations:** 1 Naval Architecture and Marine Engineering Department, University of Michigan, Ann Arbor, MI 48109, USA; 2 ReVision Consulting, LLC., 1104 Corporate Way, Sacramento, CA 95831, USA; mirko@re-vision.net

**Keywords:** marine radar, remote sensing, ocean waves

## Abstract

Marine radars have proven to be useful for measuring ocean waves, but the accuracy of the measurements is limited by several factors including the look-angle dependence of the radar signals as well as noise in the radar data. The look-angle dependence introduces a systematic error or bias in the measurements, and noise causes a random error. This paper describes a method of combining data from multiple radar frames that is optimal in the sense of minimizing the error for a set of biased measurements with random additive noise. The results are shown experimentally to increase the correlation of the radar estimates with buoy measurements.

## 1. Introduction

Marine radar measurements of ocean waves have been described by a number of investigators using both commercial navigational radars [1,2,3,4,5,6,7,8] as well as special-purpose Doppler radars [9,10,11,12,13]. Doppler measurements have the advantage of a more direct relationship between the received signals and the wave characteristics, but are more complicated and more sensitive to noise and to the effects of wave shadowing. The signals produced by commercial navigational radars, which are essentially proportional to the backscattered power or radar cross section of the ocean surface, have the advantage of hardware simplicity but are less directly related to the wave characteristics and therefore generally require some additional information to allow quantitative wave measurements. Both types of measurements require a minimum wind speed on the order of 2–3 m/s to produce enough small-scale surface roughness to scatter microwave radiation in the backward direction.

Doppler and backscattered power signals are both modulated by the presence of longer surface waves, i.e., those with wavelengths longer than at least twice the range resolution of the radar, and it is this modulation that makes the measurement of ocean wave fields possible. However, the modulation of both backscattered power and Doppler signals depends on the angle between the wave propagation direction and the instantaneous radar look direction. The exact dependence depends on the type of signal, but the modulation goes to zero for waves propagating normally to the look direction in both cases. This fact has implications for the radar measurement of ocean waves as discussed in the following section. A method of overcoming this effect is discussed in Section 3, and experimental results are presented in Section 4.

## 2. Comparison of Radar and Buoy Measurements

Wave buoys are commonly used for measuring ocean waves, and represent the standard by which alternative measurement technologies are compared. Several different methods are actually used, with GPS measurements of the instantaneous three-dimensional position of the buoy recently overtaking older methods involving measurements of the buoy acceleration and orientation. In either case, well-developed methods are used for converting the buoy data into estimates of the wave frequency and directional spectra. Time-series measurements of the buoy elevation or heave may also be compared directly with radar measurements. Such phase-resolved comparisons are deemed to be more definitive for evaluating the capability of radar for predicting ship motions or for adjusting wave energy devices, although comparisons of the directional spectrum are also of interest.

The look-directional dependence of radar measurements referred to in the previous section poses a problem for phase-resolved comparisons of radar and buoy measurements, especially in the case of a broad angular distribution or multi-modal wave field. This is because a radar measurement at a particular location or look direction cannot capture the entire wave field. If we point the radar in the direction of the buoy, the received signals will contain information about waves propagating in that direction, but will contain no information about waves propagating in the orthogonal direction. It is necessary to change the look direction to measure those waves. However, changing the look direction takes some time, and more importantly the waves measured in another direction are at a different location and will take some time to propagate to the buoy location. Thus, short-term predictions may produce better results than concurrent comparisons of radar and buoy data.

## 3. Wave Predictions Using Radar Data

Short-term wave predictions using radar data are fairly straightforward. Several methods of processing radar data exist for estimating the Fourier coefficients of the ocean surface elevation, as summarized below. Thus, predictions can be made simply by adjusting the phase of the Fourier coefficients. However, the predictions are only valid within a region down wave from the measurement region, at a distance $c_{g}T_{f}$ where $c_{g}$ is the wave group velocity and $T_{f}$ is the forecast interval. Computing the surface elevation at a given location and time may therefore require a combination of radar data collected at different times. This paper is chiefly concerned with the method of combining the data from different time intervals.

Marine radars use a narrow-beam antenna rotating at a roughly constant speed in order to produce a two-dimensional image of the ocean surface. One complete rotation forms a convenient unit of data, which is referred to as a frame. Each frame of radar data can be processed in various ways to form a set of surface elevation Fourier coefficients. Perhaps the most common way is to transform the polar format data into rectangular coordinates and perform an ordinary 2D Fourier transform on a selected rectangular subset of the image. The Fourier transform of the input data is then converted into the Fourier transform of the surface elevation using a modulation transfer function (MTF). Commonly for backscattered power data, the MTF is assumed to be a constant, although more sophisticated MTF models also exist. There are two problems with this approach, however. First, it is difficult to account for the angular dependence of the MTF since the azimuth angle varies within the analysis region. Secondly, because of this angular dependence, a single analysis region cannot adequately represent all of the waves present in the scene, particularly if there is a broad or bimodal angular distribution. These problems were the motivation for the development of the polar Fourier transform method [14], which calculates the Fourier coefficients for the direction $\phi_{n}$ from a set of input samples centered on this direction. This not only eliminates the need for a polar to rectangular coordinate transformation, but also allows the angular dependence of the MTF to be easily accounted for, and automatically selects the optimum set of input samples for the calculation of each Fourier coefficient.

In any case, the surface elevation Fourier coefficients can be denoted by the symbol $a_{mn}$ where *m* is the frame number and *n* is an index for the two-dimensional wavenumber with magnitude $k_{n}$ and direction $\phi_{n}$. The phases of the complex Fourier coefficients are shifted to a common reference location $\left(x_{m},y_{m}\right)$ and time *t_m_* for each frame. The frame-to-frame change in the phase of each coefficient is used to calculate the wave frequency, and the sign of the frequency is used to discriminate between approaching and receding waves, with negative frequencies corresponding to receding waves. This leads to a definition of $\phi_{n}$ as the opposite of the usual wave propagation direction.

Given these definitions, the surface elevation at the location (x,y) and time t can be calculated from the coefficients for the $m^{th}$ frame as

(1) $$\eta_{m}\left(x,y,t\right)=Re\overset{N}{\underset{n=1}{\sum }}a_{mn}e^{i[k_{xn}\left(x-x_{m}\right)+k_{yn}\left(y-y_{m}\right)+\omega_{n}\left(t-t_{m}\right)]}$$

where $k_{xn}=k_{n}\sin\phi_{n}$, $k_{yn}=k_{n}\cos\phi_{n}$, and $\omega_{n}$ is the frequency from the gravity wave dispersion relation, i.e., $\omega_{n}=\sqrt{gk_{n}}$ (where g is the gravitational acceleration) for deep water waves. It might be supposed that the surface elevation would be calculated most accurately at the time of the data collection, i.e., at $t=t_{m}$. However, this is not the case, at least for waves with a broad or bimodal angular distribution. This effect is illustrated by simulations in [14] where it is shown that the surface elevation at the radar location is reconstructed more accurately at $t=t_{m}+60$ s than at $t=t_{m}$ for the case considered there. This is because the wave components propagating in different directions, which are measured at different locations, require a few tens of seconds to converge at a given location near the ship.

For noisy data, it is to be expected that better estimates should be obtainable by combining the data from multiple frames, but it is not obvious how the data should be combined. Since some frames produce better estimates than others, a simple average might be less accurate than the best single frame. To approach a solution to this problem, consider a simpler case in which we have a set of *n* measurements $m_{i}$ of the same quantity  q, but each contains a scaling error as well as additive noise, i.e.,

(2) $$m_{i}=w_{i}q+n_{i}\;$$

where $w_{i}$ is a scale factor and $n_{i}$ is a zero-mean, uncorrelated random variable with the same variance for each measurement, i.e., $\langle n_{i}\rangle =0$ and $\langle n_{i}n_{j}\rangle =n^{2}\delta_{ij}$. An optimal estimate of q can be obtained by forming the linear combination of these measurements

(3) $$\hat{q}=\overset{N}{\underset{i=1}{\sum }}c_{i}m_{i}\;$$

where the coefficients $c_{i}$ are chosen to minimize the error $\varepsilon =\langle {\left(\hat{q}-q\right)}^{2}\rangle ,\;$i.e., to make

(4) $$\frac{\partial \varepsilon }{\partial c_{j}}=2\langle \left(\hat{q}-q\right)\frac{\partial \hat{q}}{\partial c_{j}}\rangle =2\langle \left(\hat{q}-q\right)m_{j}\rangle =0\;$$

or

(5) $$\langle \hat{q}m_{j}\rangle =q\langle m_{j}\rangle .$$

The left-hand side of this equation is

(6) $$\langle \hat{q}m_{j}\rangle =\overset{N}{\underset{i=1}{\sum }}c_{i}\langle m_{i}m_{j}\rangle =q^{2}w_{j}\overset{N}{\underset{i=1}{\sum }}c_{i}w_{i}+n^{2}c_{j}\;$$

and the right-hand side is

(7) $$q\langle m_{j}\rangle =q^{2}w_{j}.$$

Combining (5)–(7), we have

(8) $$\;c_{j}=\frac{q^{2}}{n^{2}}\left(1-\overset{N}{\underset{i=1}{\sum }}c_{i}w_{i}\right)\;w_{j}.\;$$

This equation has the solution

(9) $$c_{j}=w_{j}\;{\left(\overset{N}{\underset{i=1}{\sum }}w_{i}^{2}+\frac{n^{2}}{q^{2}}\right)}^{-1}\;$$

which can be verified by substitution of (9) into (8).

For the wave prediction case, we consider each Fourier coefficient $a_{mn}$ as a measurement of a wave component having a scale factor $w_{mn}$ that depends on the location of the measurement relative to the radar. In the simplest case, we may set $w_{mn}=1\;$if the measurement is made within a distance $r_{2}$ of the radar, where $r_{2}$ is the maximum range selected for processing and $w_{mn}=0$ if the origin of the wave component is outside of this measurement region. The location of the measurement is calculated by tracing each wave component backward from the location (x,y) and time t to the time $t_{m}$ using the group velocity of that component. Thus, the distance from the radar is given by

(10) $$r_{mn}=\sqrt{{\left[\left(x-x_{m}\right)-c_{xn}\left(t-t_{m}\right)\right]}^{2}+{\left[\left(y-y_{m}\right)-c_{yn}\left(t-t_{m}\right)\right]}^{2}}$$

where $c_{xn}$ and $c_{yn}$ are the *x* and *y* components of the wave group velocity corresponding to the wavenumber $\left(k_{n},\phi_{n}\right)$. The scale factor for this wave component is then

(11) $$w_{mn}=\{\begin{array}{c} 0\;for\;\;r_{mn}>r_{2}\;\\ 1\;for\;r_{mn}<r_{2} \end{array}\;$$

in the simplest case, or $w_{mn}=f\left(r_{mn}\right)$ in general, where f(r) could include a tapering function and/or a minimum range $r_{1}\;$due to the near-range dead zone of the radar. The surface elevation can then be calculated from *M* frames of radar data as

(12) $$\eta \left(x,y,t\right)=Re\overset{M}{\underset{m=1}{\sum }}\overset{N}{\underset{n=1}{\sum }}a_{mn}c_{mn}e^{i[k_{xn}\left(x-x_{m}\right)+k_{yn}\left(y-y_{m}\right)+\omega_{n}\left(t-t_{m}\right)]}\;$$

where

(13) $$\;c_{mn}=w_{mn}\;{\left(\overset{M}{\underset{\mu =1}{\sum }}w_{\mu n}^{2}+1/S_{mn}\right)}^{-1}.$$

Here $S_{mn}={\left|a_{mn}\right|}^{2}/\alpha_{n}^{2}\;$is the signal-to-noise ratio for this sample, $\alpha_{n}^{2}$ being an estimated spectral noise level.

## 4. Experimental Results

**Figure 1:**
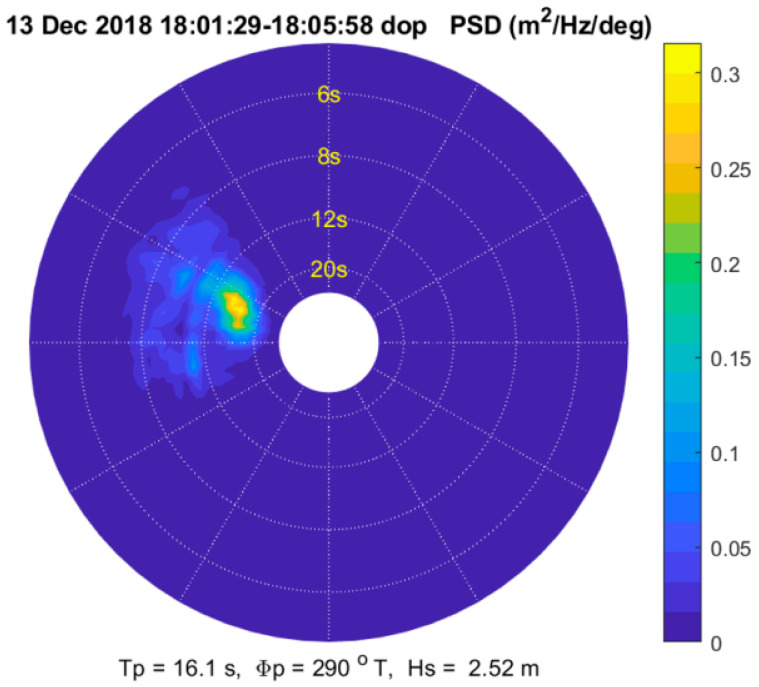
Directional wave spectrum computed from Doppler radar data.

Results are presented in this section for a dataset collected in the Pacific Ocean near Santa Cruz, California on 13 December 2018, as described in [15]. The radar used in this experiment was a Koden X-band navigational radar modified to collect coherent (Doppler) data as described in [16]. The relevant radar and antenna parameters are shown in Table 1. The antenna was mounted on a research vessel at a height of 8 m above the water surface, and the data were recorded with a 16-bit analog-to-digital converter at a rate of 80 MSPS. The dataset analyzed here consists of a 5-min segment of radar data collected within about 60 m of a directional wave buoy. The wind speed was approximately 3 m/s and the significant wave height was about 2 m. Waves were predominantly from the WNW with a peak period of about 15 sec. A plot of the directional wave spectrum inferred from the radar data is shown in Figure 1.

Fourier coefficients were estimated from the radar data as described in [14,15]. Briefly, the polar Fourier transform (PFT) of the Doppler velocity data is scaled by an appropriate factor to convert the discrete PFT samples into surface elevation Fourier coefficients. The Fourier coefficients were used to calculate the surface elevations from a single frame using Equation (1) and from multiple frames using Equation (12). The radar-derived and buoy-measured surface elevations are plotted versus time for several cases and the comparisons are summarized in the form of correlation coefficients and mean absolute errors for each case considered.

**Figure 2:**
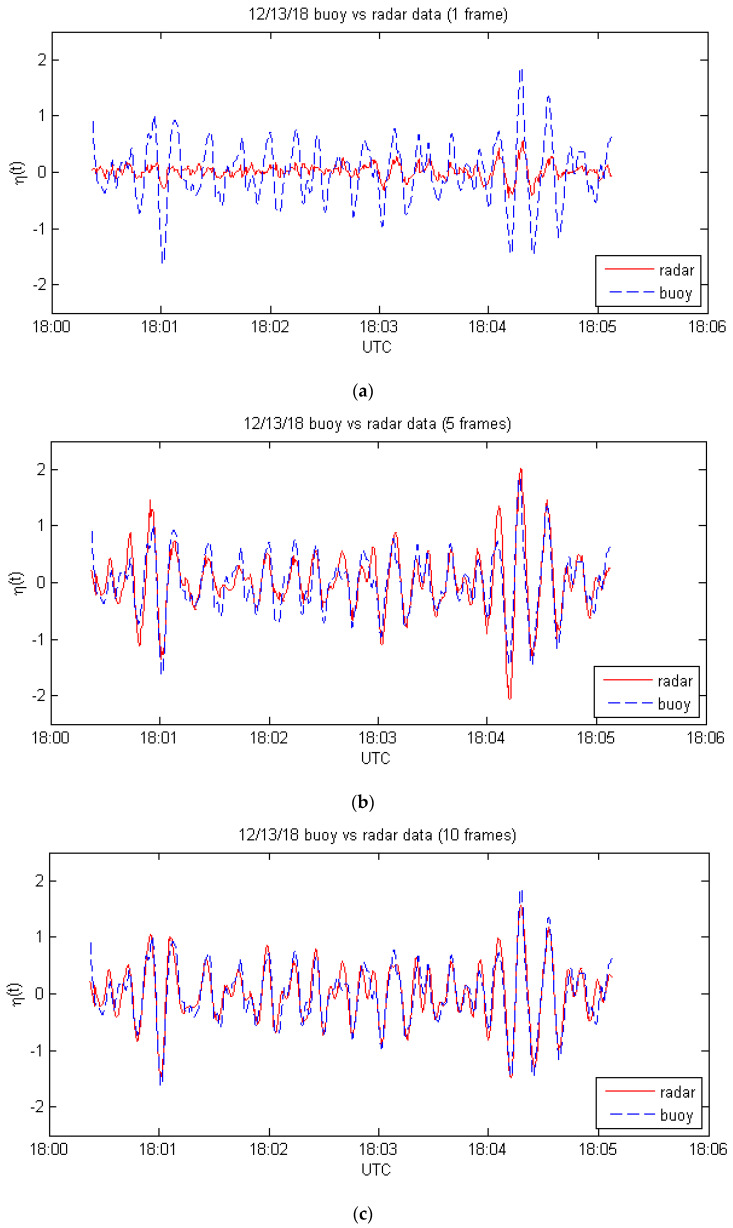
Comparison of buoy measurements with nearly concurrent radar estimates for: (**a**) one frame, from Equation (1); (**b**) 5 frames, from Equation (12); (**c**) 10 frames, from Equation (12).

The buoy data were recorded at a rate of 5 samples per second, and the duration of each radar frame is approximately 2.7 s for this dataset, so there are 13 or 14 buoy samples within each radar frame. In the first case, Equation (1) was evaluated at the buoy location and at the time of each buoy sample using the Fourier coefficients from the nearest radar frame. The resulting surface elevations are compared with the buoy data in Figure 2a.

In the second case, the surface elevation was calculated for each buoy sample from (12) using the Fourier coefficients from 5 frames of data ending with the frame nearest to the buoy sample under consideration. These results are shown in Figure 2b. In the third case, the same procedure was used, but with 10 frames of data. The results for this case are shown in Figure 2c. The correlation coefficients and mean absolute errors for each case are shown in Table 2.

The accuracy of the radar estimates is dramatically improved in this set of cases when multiple-frame processing is used. Part of the reason for this is that the buoy is close to the near-range dead zone of the radar. Thus, radar measurements made slightly earlier than the buoy measurements allow for the measured waves to propagate to the buoy location. Nevertheless, the processing method does appear to combine the information from multiple frames successfully.

**Figure 3:**
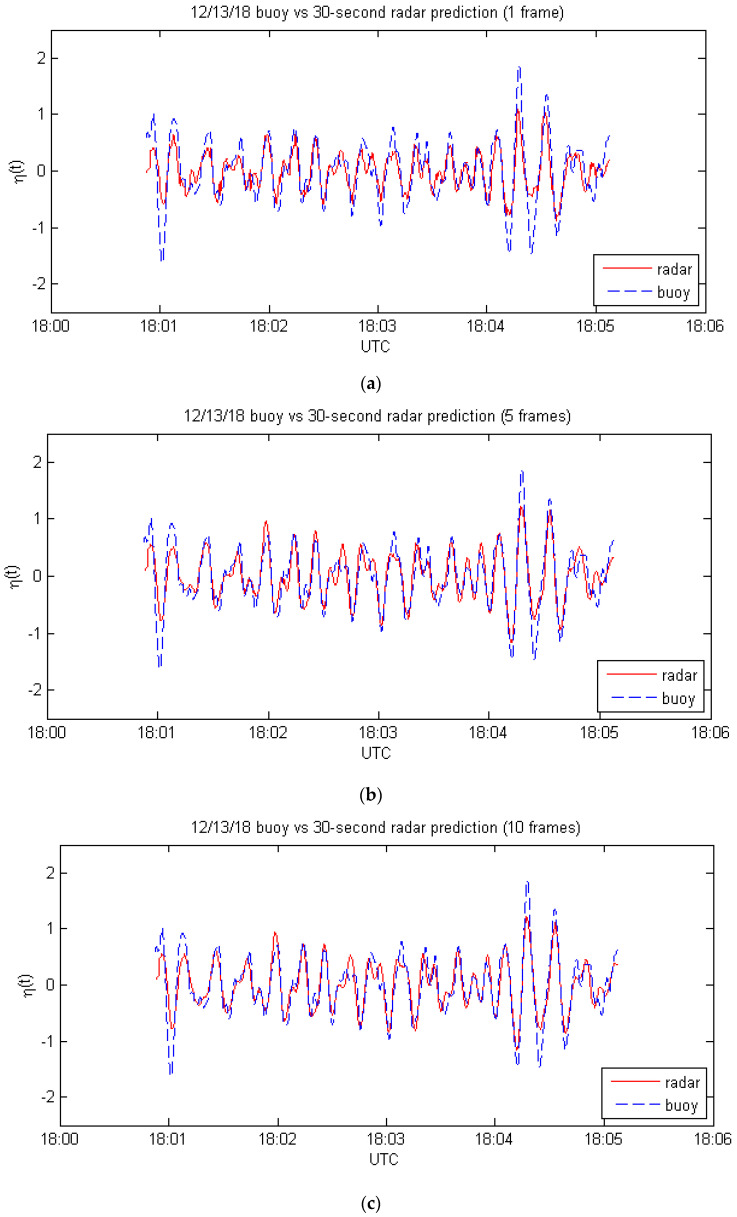
Comparison of buoy measurements with 30 s radar predictions for: (**a**) one frame, from Equation (1); (**b**) 5 frames, from Equation (12); (**c**) 10 frames, from Equation (12).

The next set of cases involves a 30 s prediction of the wave field for the same set of buoy samples. The same procedure was used, except that calculations were made for times 30 s later than the last radar frame used. The results are shown in Figure 3 and Table 3.

The 30 s predictions show a significant but not as dramatic improvement with the number of frames used. The reason appears to be that the 30 s prediction uses measurements within the “sweet spot” of the radar, so the multiplicative bias is quite small and the signal-to-noise ratio is large for all of the frames used.

**Figure 4:**
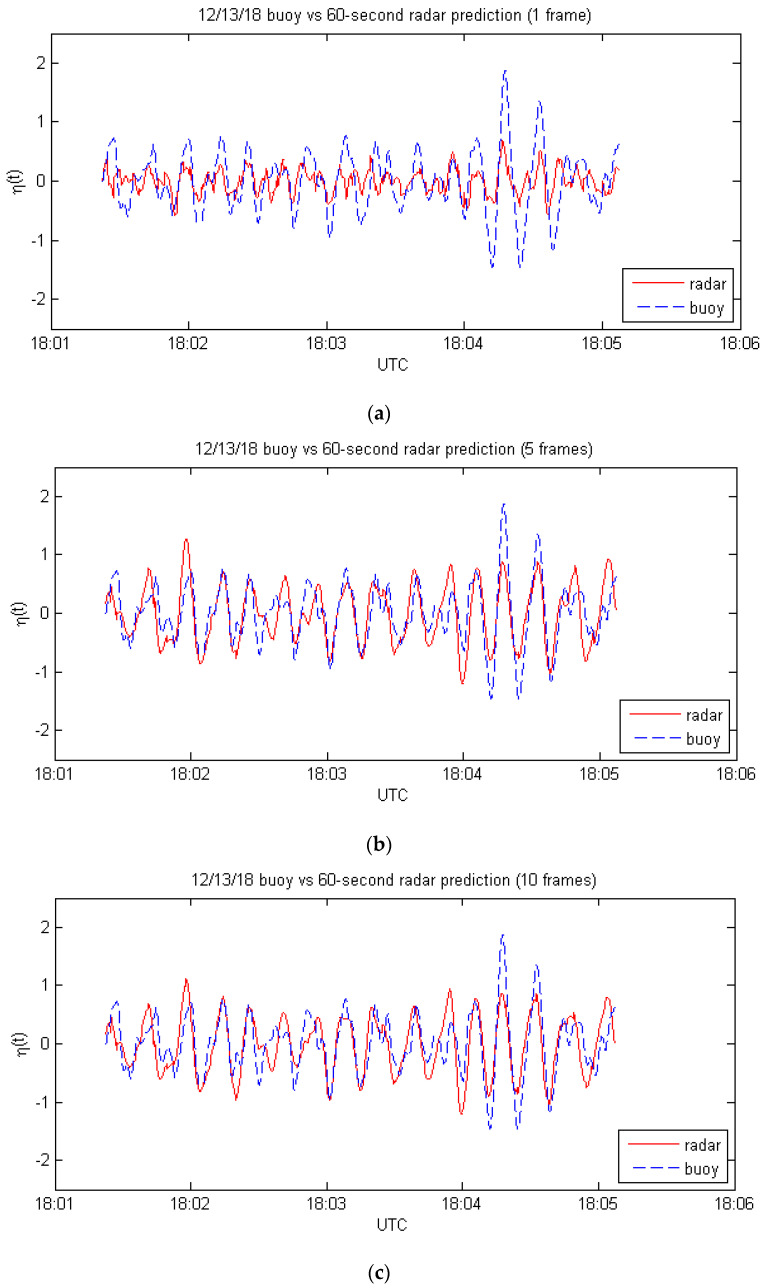
Comparison of buoy measurements with 60 s radar predictions for: (**a**) one frame, from Equation (1); (**b**) 5 frames, from Equation (12); (**c**) 10 frames, from Equation (12).

The final set of cases is the same as the previous one except that the forecast horizon is 60 s instead of 30 s. The results are shown in Figure 4 and Table 4.

The 60 s predictions are not as good as the 30 s ones because they require measurements at the far end of the useable range of the radar. There is a significant improvement in the 5-frame results as compared to the single-frame predictions, but going from 5 frames to 10 frames produces very little change, presumably because the scale factors ($w_{mn}$ values) are very small for the additional frames.

## 5. Discussion

The procedure described in this paper for combining the data from multiple radar frames appears to be effective for improving the wave field estimates from marine radar data. The method can reduce the effects of noise and slightly enlarge the space-time regions over which predictions can be made. However, the forecast horizons for radar predictions are still limited to some tens of seconds by the maximum range at which radar measurements can be made. For Doppler radars, this maximum range is influenced somewhat by the wind speed and radar power, but is ultimately limited by wave shadowing effects. These effects can be reduced by increasing the antenna height, but this height is of course constrained by practical considerations. Backscattered power measurements as provided by conventional navigational radars are not limited in the same way by wave shadowing effects, and longer forecasts can be anticipated but additional information may be required for quantitative predictions.

## Figures and Tables

**Table 1:** Doppler radar parameters.

peak transmit power	25 kW
pulse length	80 ns
pulse repetition frequency	2 kHz
antenna polarization	vertical
antenna beamwidth	1.8 deg

**Table 2:** Error statistics for nearly concurrent radar and buoy measurements.

*M*	Correlation Coefficient	Mean Absolute Error (m)
1	0.665	0.362
5	0.877	0.215
10	0.927	0.158

**Table 3:** Error statistics for 30 s radar predictions versus buoy measurements.

*M*	Correlation Coefficient	Mean Absolute Error (m)
1	0.858	0.231
5	0.899	0.184
10	0.912	0.171

**Table 4:** Error statistics for 60 s radar predictions versus buoy measurements.

*M*	Correlation Coefficient	Mean Absolute Error (m)
1	0.576	0.340
5	0.731	0.302
10	0.738	0.299

## Data Availability

The radar data sets used in this study are not publicly available.
